# Supplementary material for: Test-retest reliability of behavioral and computational measures of advice taking under volatility
Source: PLoS One. 2024 Nov 18;19(11):e0312255. doi: 10.1371/journal.pone.0312255 (PMC11573178; doi:10.1371/journal.pone.0312255)
Supplement: S1 Appendix — (PDF) [file pone.0312255.s001.pdf]

Supplementary materials:
Test-retest reliability of behavioral and computational measures of advice taking under volatility

1 Model recovery and model selection

Just like Hauke et al. (2024), we found that that the control model 1b could not be well recovered. All other models - and most importantly, the primary models of interest (model 1 and model 2) - were recoverable with high probability (**Supplementary Fig 1**).

Random-effects Bayesian model selection revealed that model 2 was the winning model for both session 1 and session 2 (**Supplementary Fig 2**).

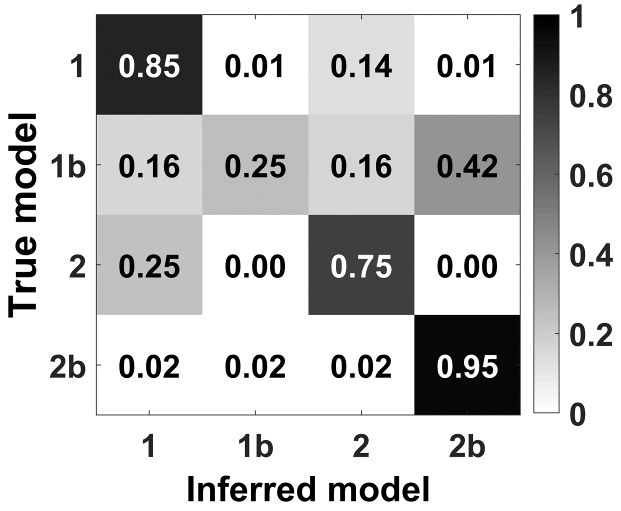

Supplementary Figure 1: **Model recovery.** The confusion matrix shows average protected exceedance probability: averaged across 20 data sets simulated with the same parameters but with different random seeds to account for the stochasticity effects.

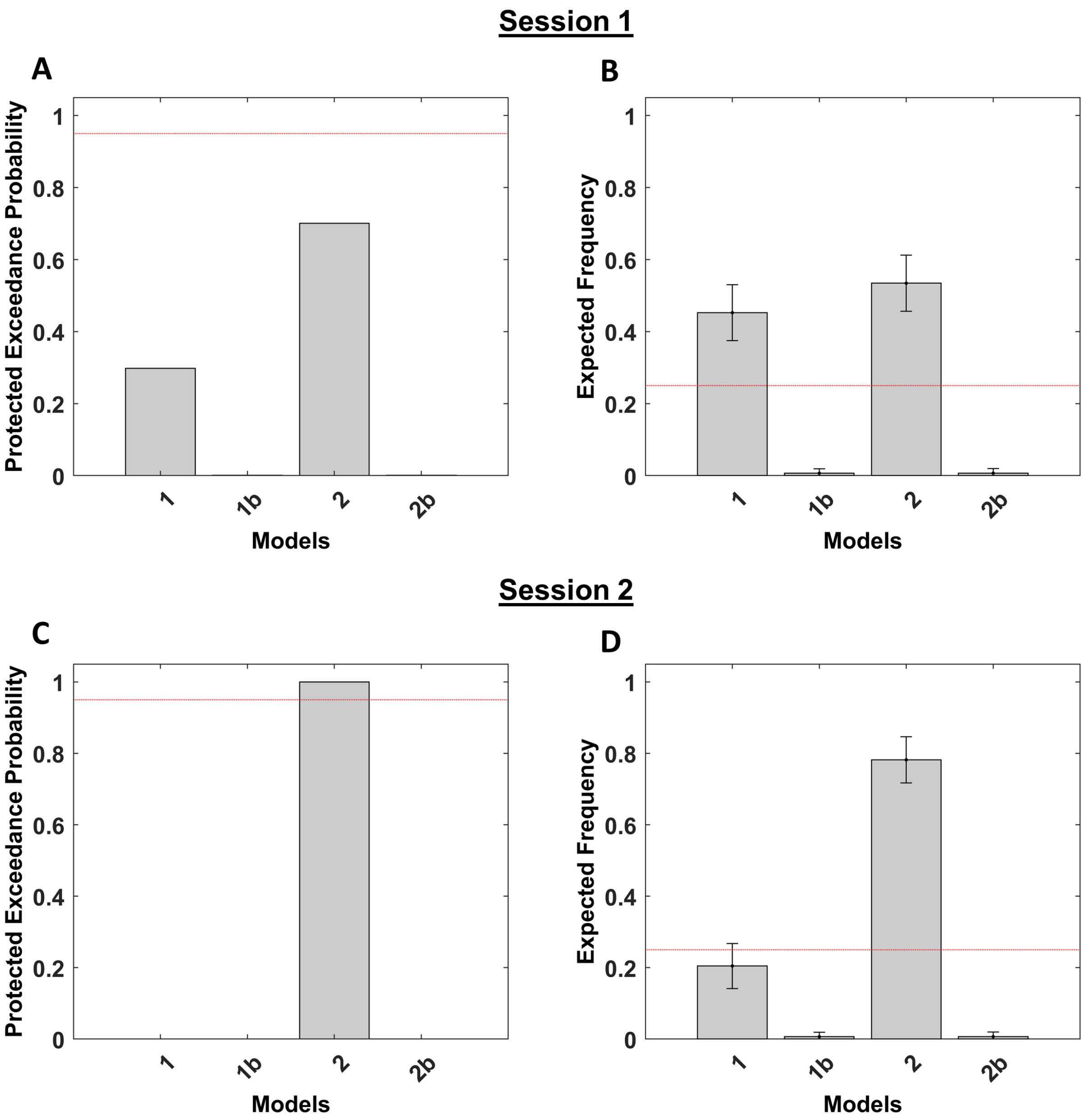

Supplementary Figure 2: **Random-effects Bayesian Model Selection results.** Models: standard HGF with free parameters (1) or Bayes Optimal parameters (1b) and mean-reverting HGF with free parameters (2) or Bayes Optimal parameters (2b). In both testing sessions model 2 is the winning model.

2 Test-retest reliability results after removing influential data points

The visual inspection of the scatter plot of κ_2 test-retest reliability suggested that some data points, while not being extreme univariate outliers may nonetheless have exerted outsized influence on the estimated reliability. To verify this, we used Cook's distance (Cook, 1977) to remove the most influential data points affecting a linear regression model ($x_{T1} \sim x_{T2}$). We used the standard threshold of 4/N, where N is the number of data points, which in our case was 39. This indeed resulted in much better test-retest reliability results for κ_2 (**Supplementary Fig 3D**), improving it from 0.04 to 0.40. For completeness we also report how this analysis affected other parameters, although the changes were less noteworthy (**Supplementary Fig 3**).

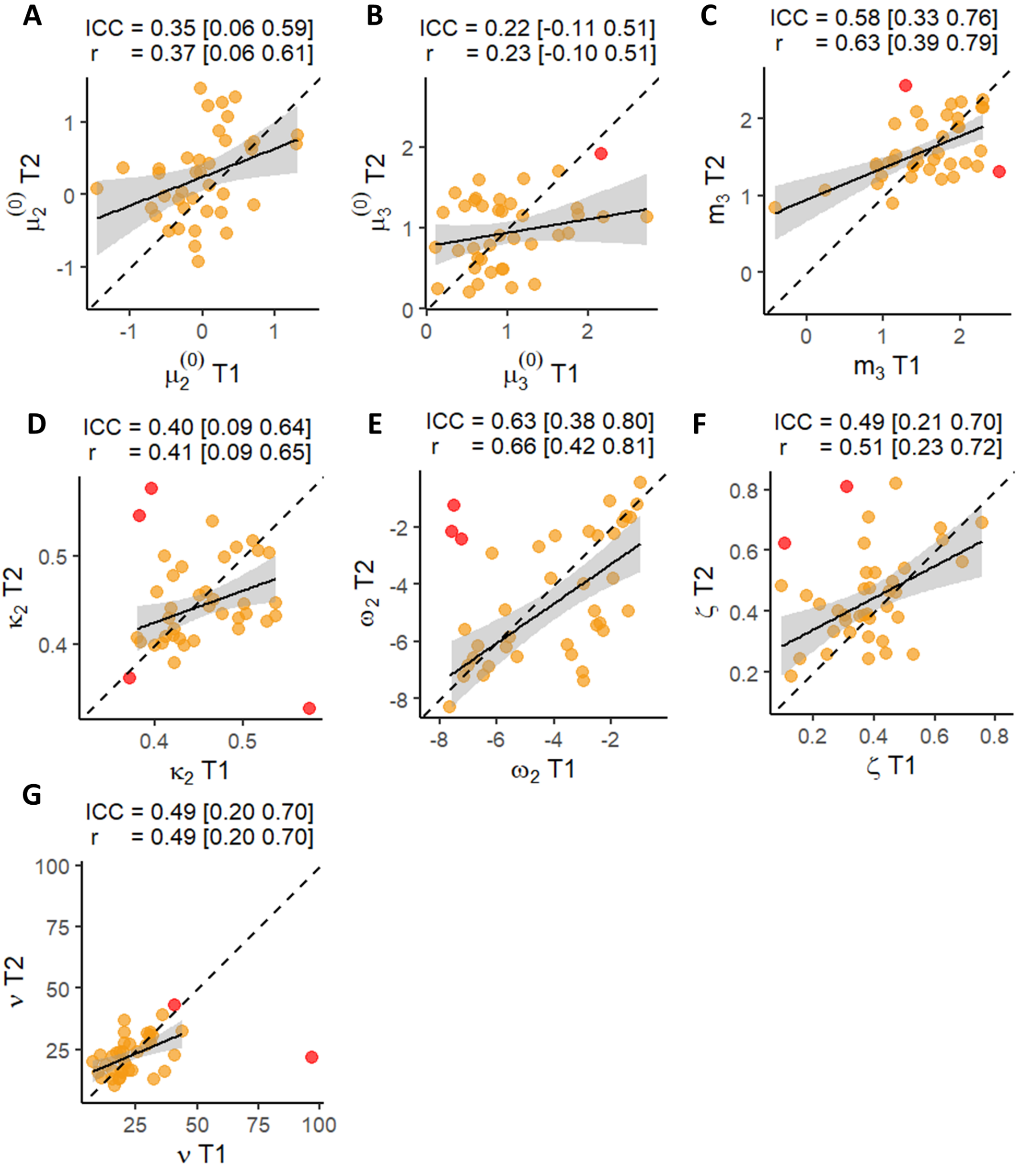

Supplementary Figure 3: **Test-retest reliability of model 2 parameters after removing data points with outsized influence, as determined using Cook's distance..**

3 Parameter recovery and test-retest reliability of model 1

To make sure that our results about the reliability of computational measures did not hinge on the winning model only, we also assessed model 1. Given that model 1 is less complex (it does not involve the drift at the 3rd level and has 6 instead of 7 free parameters), we expected it might fare better in terms of reliability. However, the results were rather similar: test-retest reliability was poor ($ICC < 0.5$) for most parameters, even when recoverability was very high (**Supplementary Fig 4**)

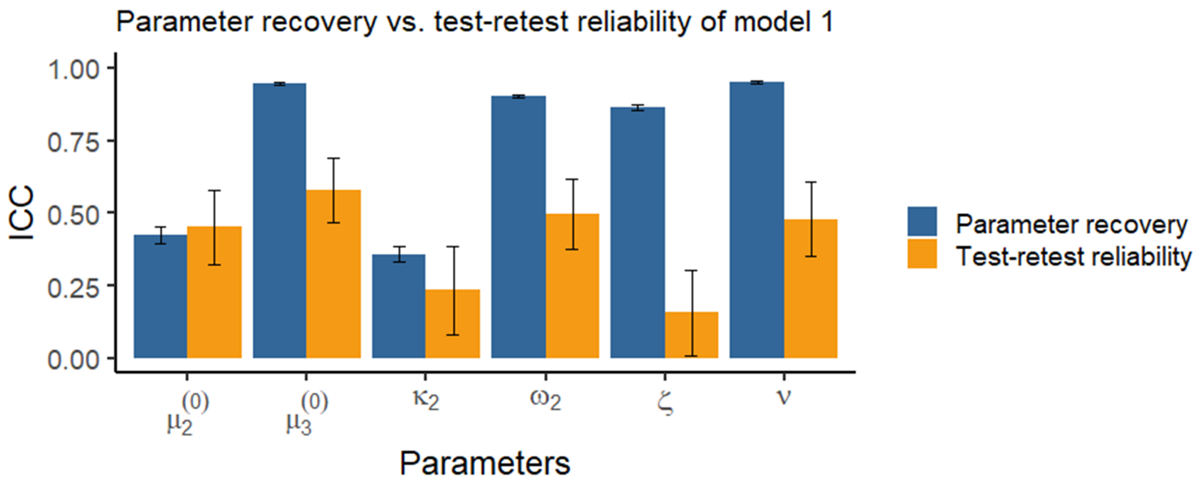

Supplementary Figure 4: **Parameter recovery vs. test-retest reliability of model 1 parameters.**.

4 Fixing parameters to test for collinearity

To investigate if collinearity was the reason why parameters $\mu_2^{(0)}$, $\mu_3^{(0)}$, and κ_2 in model 2 had low recoverability, we refit the model to the data by fixing each of these parameters at a time and observing how it affected the recoverability of the remaining parameters - if collinearity was the issue, fixing one of the parameters would improve the recovery of the remaining ones. We followed the same fitting procedure as described in the main text and fixed the parameters to the corresponding priors as detailed in **Table 1** in the main text. We did not observe any major improvements after fixing $\mu_2^{(0)}$ (**Supplementary Fig 5A**), or fixing κ_2 (**Supplementary Fig 5B**), or even fixing both of these parameters at once (**Supplementary Fig 5C**). Interestingly, fixing all three parameters worsened the recoverability of parameters ν and m_3 (**Supplementary Fig 5D**).

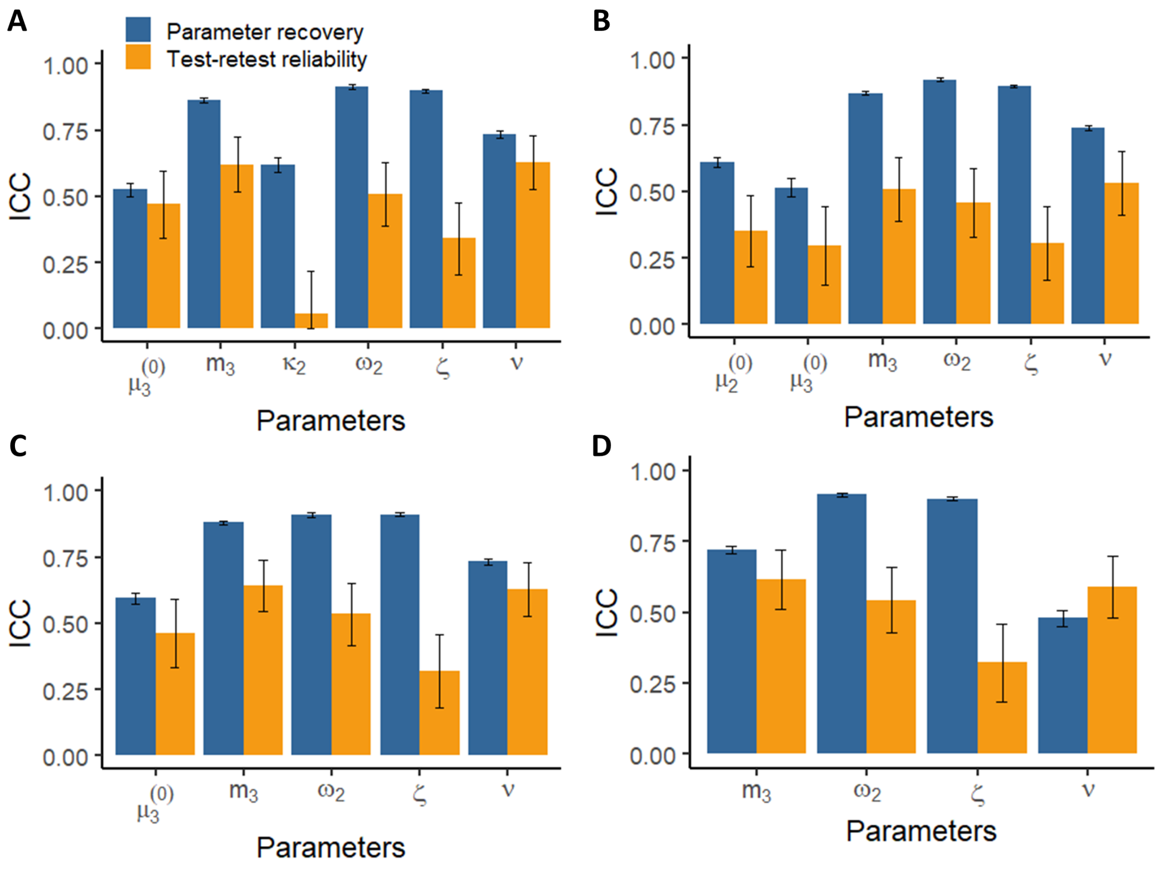

Supplementary Figure 5: **Parameter recovery vs. test-retest reliability for reduced versions of model 2, to test for potential collinearity.** (**A**) Fixed $\mu_2^{(0)}$. (**B**) A model with fixed κ_2. (**C**) Fixed $\mu_2^{(0)}$ and κ_2. (**D**) Fixed $\mu_2^{(0)}$, κ_2, and $\mu_3^{(0)}$.

5 EEG vs fMRI sessions

To assess if test-retest reliability might have been affected by different environments, we compared parameter estimates between EEG and fMRI sessions (the order of which were randomized across participants). We found substantial evidence ($BF_{01} > 3$) that neither of the parameters were systematically affected by different neuroimaging environments (**Supplementary Fig 6**).

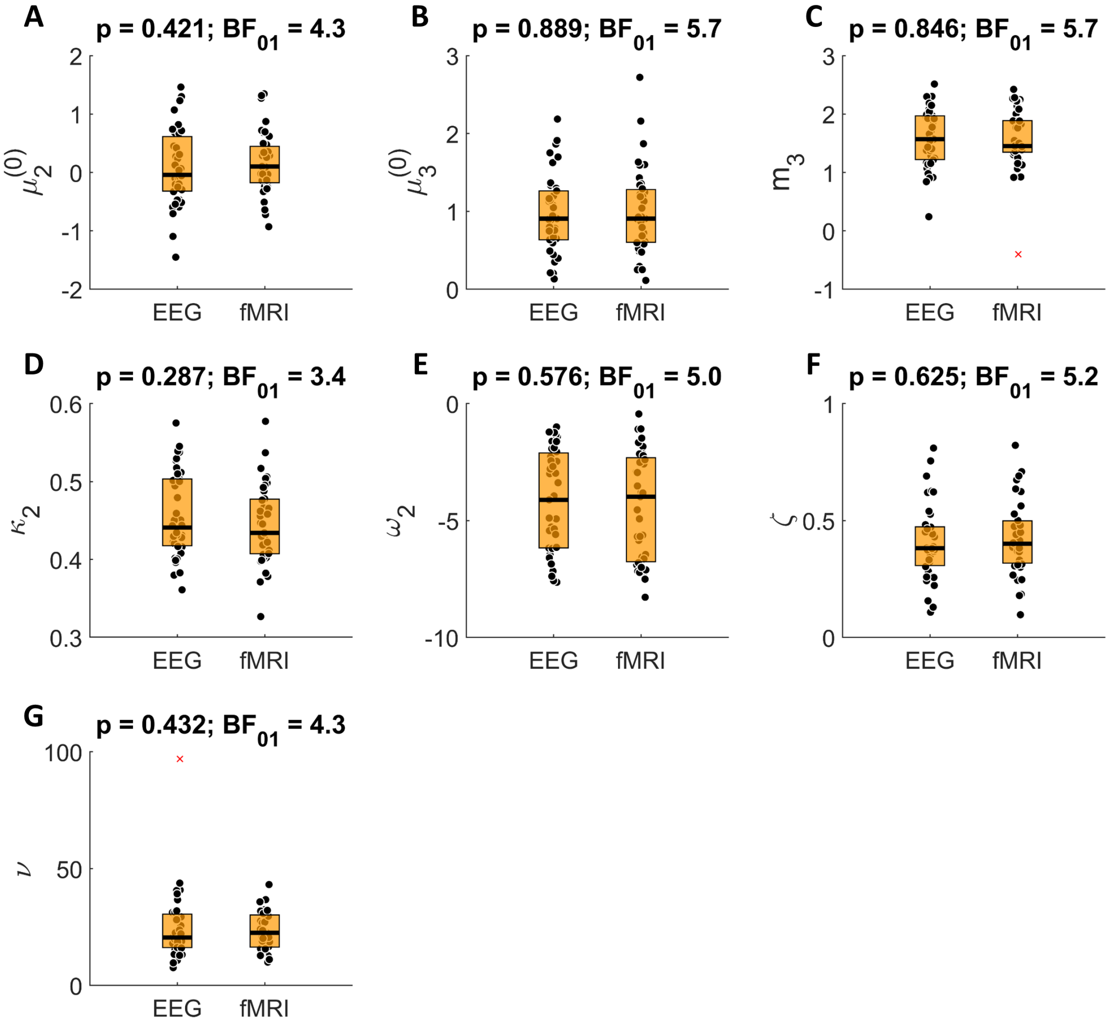

Supplementary Figure 6: **Parameter estimates from EEG and fMRI sessions.** (**A**) Prior expectations about adviser's fidelity before starting the task (**B**) Prior expectations about adviser's volatility before starting the task. (**C**) Volatility equilibrium point (**D**) Phasic learning rate about adviser's fidelity (**E**) Tonic learning rate about adviser's fidelity (**F**). The relative weighing of the advice compared to the non-social cue. (**G**) Decision noise. Red crosses indicate outliers. p-values and Bayesian factors for the null hypothesis (BF_{01}) of paired t-tests are presented above each plot.

6 Parameter recovery vs test-retest reliability in previous studies

Not many studies assessing test-retest reliability also report parameter recovery results (Karvelis et al., 2023). In such cases it is difficult to know which sources of variance are primarily responsible for low test-retest reliability (e.g., measurement error or within-subject variability). Even when parameter recovery results are reported, they tend to be not fully utilized to disambiguate the different sources of variance. We pooled the data from such studies and put them together in a single plot to gain more insight (**Supplementary Fig 7**).

First, we find that all studies report considerably high parameter recoverability (> 0.75). Second, test-retest reliability tends to always be considerably lower than parameter recoverability. This difference also tends to increase with longer intervals between test and retest.

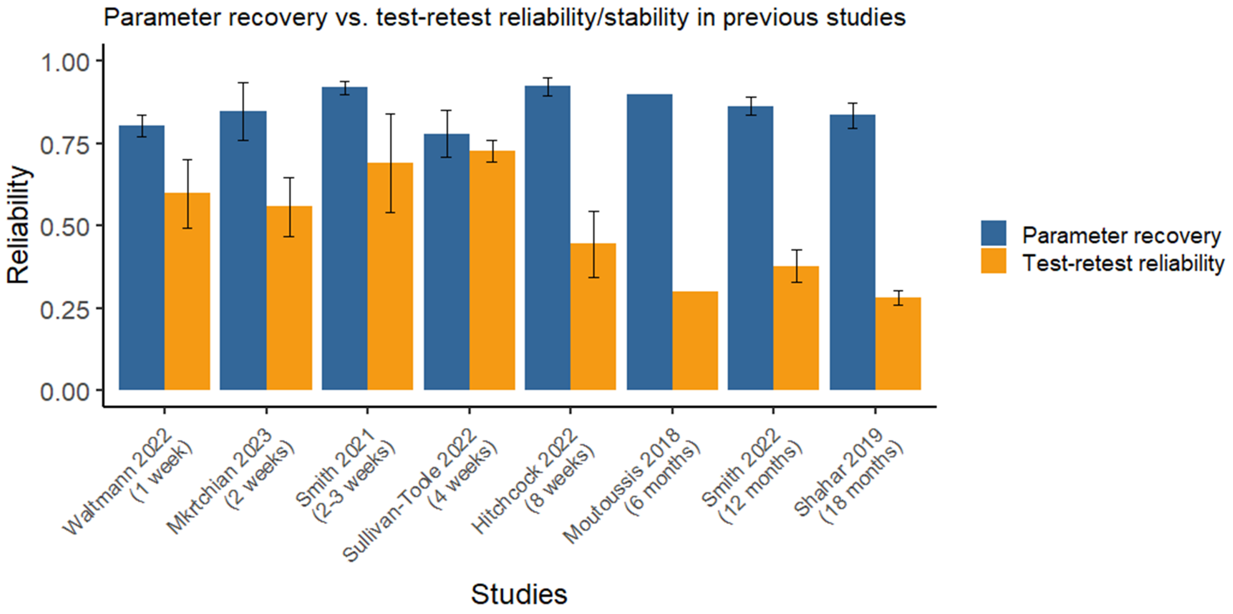

Supplementary Figure 7: **The differences in parameter recovery and test-retest reliability of parameter estimates in previous studies.** The studies are sorted by the length of the interval between test and retest (indicated within the parentheses): Waltmann et al. (2022); Mkrtchian et al. (2023); Smith et al. (2021); Sullivan-Toole et al. (2022); Hitchcock et al. (2022); Moutoussis et al. (2018); Smith et al. (2022); Shahar et al. (2019). Each column represents reliability averaged across all parameter estimates; the error bars indicate standard error. Note that some studies used ICC, some used Pearson's correlation coefficient, and some used Spearman's correlation coefficient to assess test-retest reliability and parameter recoverability - here we ignore the differences between these metrics and plot them along the same Y-axis.

7 Test-retest reliability of model 2 parameters when using the same number of trials as in Hauke et al. (2024)

To be able to more directly relate our results to the findings of Hauke et al. (2024), we reran the test-retest analysis using the same number of trials (136 instead of 153). Perhaps unsurprisingly, we found that test-retest reliability of model parameters was very similar to that reported in the main text (**Supplementary Fig 8**).

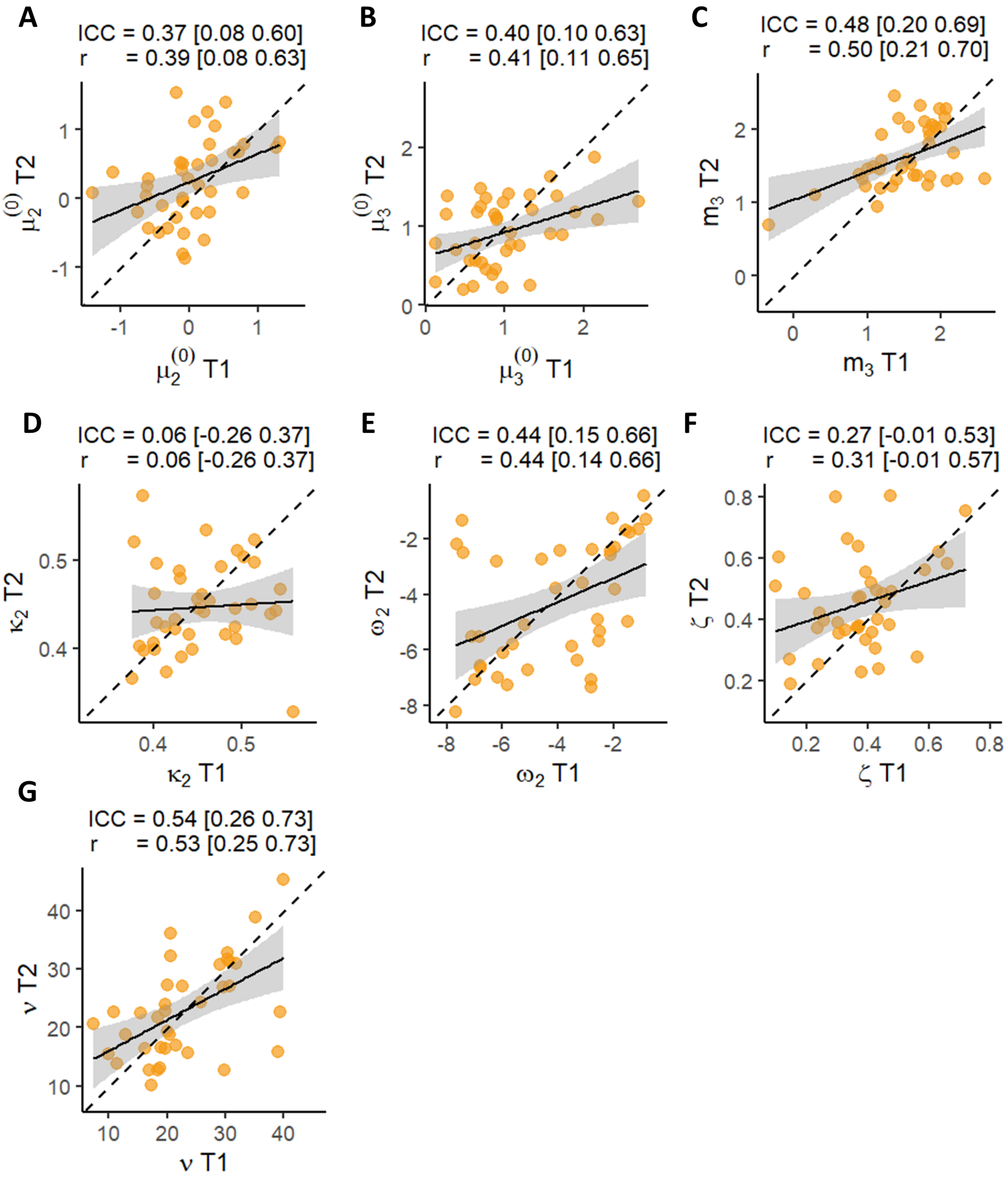

Supplementary Figure 8: **Test-retest reliability of model 2 using the same number of trials (136) as in Hauke et al. (2024)** (**A**) Prior expectations about adviser's fidelity before starting the task (**B**) Prior expectations about adviser's volatility before starting the task. (**C**) Volatility equilibrium point (**D**) Phasic learning rate about adviser's fidelity (**E**) Tonic learning rate about adviser's fidelity (**F**). The relative weighing of the advice compared to the non-social cue. (**G**) Decision noise. ICC(A,1) and Pearson's correlation coefficients are shown above each panel. The square brackets indicate 95% confidence intervals.

References

Cook, R. D. (1977). Detection of influential observation in linear regression. *Technometrics*, 19(1):15–18.

Hauke, D. J., Wobmann, M., Andreou, C., Mackintosh, A. J., de Bock, R., Karvelis, P., Adams, R. A., Sterzer, P., Borgwardt, S., Roth, V., et al. (2024). Altered perception of environmental volatility during social learning in emerging psychosis. *Computational Psychiatry*, 8(1):1.

Hitchcock, P. F., Britton, W. B., Mehta, K. P., and Frank, M. J. (2022). Self-judgment dissected: A computational modeling analysis of self-referential processing and its relationship to trait mindfulness facets and depression symptoms. *Cognitive, Affective, & Behavioral Neuroscience*, pages 1–19.

Karvelis, P., Paulus, M. P., and Diaconescu, A. O. (2023). Individual differences in computational psychiatry: a review of current challenges. *Neuroscience & Biobehavioral Reviews*, page 105137.

Mkrtchian, A., Valton, V., and Roiser, J. P. (2023). Reliability of decision-making and reinforcement learning computational parameters. *Computational Psychiatry*, 7(1):30–46.

Moutoussis, M., Bullmore, E. T., Goodyer, I. M., Fonagy, P., Jones, P. B., Dolan, R. J., Dayan, P., and in Psychiatry Network Research Consortium, N. (2018). Change, stability, and instability in the pavlovian guidance of behaviour from adolescence to young adulthood. *PLoS computational biology*, 14(12):e1006679.

Shahar, N., Hauser, T. U., Moutoussis, M., Moran, R., Keramati, M., Consortium, N., and Dolan, R. J. (2019). Improving the reliability of model-based decision-making estimates in the two-stage decision task with reaction-times and drift-diffusion modeling. *PLoS computational biology*, 15(2):e1006803.

Smith, R., Kirlic, N., Stewart, J. L., Touthang, J., Kuplicki, R., McDermott, T. J., Taylor, S., Khalsa, S. S., Paulus, M. P., and Aupperle, R. L. (2021). Long-term stability of computational parameters during approach-avoidance conflict in a transdiagnostic psychiatric patient sample. *Scientific reports*, 11(1):1–13.

Smith, R., Taylor, S., Stewart, J. L., Guinjoan, S. M., Ironside, M., Kirlic, N., Ekhtiari, H., White, E. J., Zheng, H., Kuplicki, R., et al. (2022). Slower learning rates from negative outcomes in substance use disorder over a 1-year period and their potential predictive utility. *Computational Psychiatry*, 6(1).

Sullivan-Toole, H., Haines, N., Dale, K., Olino, T., et al. (2022). Enhancing the psychometric properties of the iowa gambling task using full generative modeling. *Computational Psychiatry*, 6(1):12=89–212.

Waltmann, M., Schlagenhauf, F., and Deserno, L. (2022). Sufficient reliability of the behavioral and computational readouts of a probabilistic reversal learning task. *Behavior Research Methods*, pages 1–22.
