# Supplementary material for: Test-retest reliability of behavioral and computational measures of advice taking under volatility
Source: PLoS One. 2024 Nov 18;19(11):e0312255. doi: 10.1371/journal.pone.0312255 (PMC11573178; doi:10.1371/journal.pone.0312255)
Supplement: Attachment — Submitted filename: Response to reviewers.docx [file pone.0312255.s002.docx]

**“Test-retest reliability of behavioral and computational measures of advice taking under volatility”**

**Responses to reviewers**

We would like to thank the reviewers for taking the time to provide thoughtful comments on our manuscript. We believe that the revisions in response to the reviewers’ comments really helped to make the analysis more robust and the paper more accessible.

Below we provide answers to these comments and detail the associated revised parts of the manuscript. Please find all reviewer comments in *italics.* All author responses are in plain text while revisions are highlighted in **bold**.

*Reviewer #1: The paper covers a topic of significant importance and had been written very well. The extensive statistical analysis and depth covered by statistical descriptions provides for an interesting and informative read. Even though, based on my level of understanding, there were no technical issues that require corrections, addressing the below noted possible writing discrepancies would make the paper even better:*

*• Page 2, Line 68: Would it be more appropriate to start the sentence with a word rather than a numerical text? Four as opposed to 4.*

Thank you for spotting this, the sentence now starts with ‘Four’.

*• Page 2: It appears that the task of the participants involves a total of 153 trials. The text of the methodology section does not seem to specifically mention this. However, it could be inferred from details from different parts of the text (e.g. page 6) as well as Figure 1B. It would be better if this was specifically noted under section 2.3.*

Thank you for spotting this. In section 2.3 we now explicitly state: “**In total, there were 153 trials**.”

*• Page 2, Line 94: The word ‘they’ seem to be repeated.*

Thank you for spotting this, it has now been corrected.

*• Page 4, Equation 4: It seems the ‘x’ shown as a subscript should be ‘3’.*

Thank you for spotting this, it has now been corrected.

*• Page 4, Equation 5: Again, it seems the ‘x’ shown as a subscript should be ‘3’.*

Thank you for spotting this, it has now been corrected.

*• Page 5, Line 150: I wonder if the two models are named in the wrong order, considering how they are discussed later in the paper? Shouldn’t Eq. 5 HGF with the mean reverting process be model 2, and Eq. 4 HGF without the mean reverting process be model 1?*

Thank you for spotting this, we have now corrected section 2.4.2 to say: “In total, we have tested 2 variants of the HGF: **standard HGF, Eq. 4 (model 1), and HGF with the mean-reverting process at the third level, Eq. 5 (model 2).”**

*• Page 11: It seems Figure 7 is not referred to in the text. Perhaps it was meant to be noted at Page 10, Line 263?*

Thank you for spotting this, we now refer to it in section 3.5: “Among the computational measures, only µ(0) and ζ estimates were significantly different between the sessions (**Fig 7**).”

*• Page 11, Line 282: Should the “effects an all three” be “effects on all three”?*

Thank you for spotting this, it has now been corrected.

*• Page 12, Line 288: There is a repeated ‘validity’ term.*

Thank you for spotting this, it has now been corrected.

*Reviewer #2: This article presents evidence from N=39 subjects that the behavioural and computational measures of a task assessing advice taking under volatility have generally poor test-retest reliability, partially due to poor parameter recoverability. Given that the task and computational model have already been used to study psychiatric disorders and development, the article is relevant and timely, contributing to a growing body of research documenting the reliability of task-derived measures of interindividual differences. Clear merit notwithstanding, there are a few key points that I believe the authors should address before the manuscript is ready for publication.*

*Major:
1.Methods:
a. Participants: Is there any information on age, gender, SES or other demographics / sample characteristics? If so, this should be provided. Also, it does not become clear whether the participants were healthy controls, FEP patients, or people at high risk for psychosis.*

We have now added more details about sample characteristics in section 2.1:

“Four participants completed only a single task session, leaving 39 participants for the analysis presented in this paper. **The remaining sample included 20 female and 19 male participants, with an average age of 28.85 (SD = 4.03).**”

All participants were healthy controls, as stated in section 2.1: “participants were recruited from the general population”. However, we can see how referring to the larger study in section 2.2 might have caused some confusion. In section 2.2 we now clarify:

“In this paper, however, we will focus solely on the behavioral data, **and only on the 39 healthy controls**.”

*b. Behavioural measures: There is no description as to how behavioural measures are derived. Even if this is done by simply taking means, this should be described properly. I would also strongly encourage the use of trial-by-trial analyses using hierarchical models (see Rouder & Haaf’s 2019 paper "A psychometrics of individual differences in experimental tasks"). I understand from the discussion that the authors have qualms here – please see below for my response to those qualms.*

We have now included and additional subsection 2.4, where we describe the behavioral measures in more detail:

**“ 2.4 Behavioral measures**

**For the behavioral analysis we computed standard behavioral measures. This included: 1) Total accuracy, which was defined as the fraction of trials in which participants chose the correct response. This measure captures the overall level of performance. 2) Advice taking probability, which was defined as the fraction of trials in which participants acted in accordance with the social cue. This measure captures the tendency to rely on advice, and we computed it separately for stable and volatile phases of the task, as well as for the whole task. 3) Win-stay probability, which was defined as the fraction of trials in which participants kept the same strategy (trusting the advice or not) following a winning trial. 4) Lose-switch probability, which was defined as the fraction of trials in which participants switched their strategy (trusting the advice or not) following a losing trial. Both win-stay and lose-switch measures capture how quickly participants learn from positive and negative feedback, respectively. However, it is a rather rough proxy for the learning rate, because we expect it to dynamically change depending on how volatile the adviser’s strategy is perceived to be at any given point in the task – to capture these dynamics more explicitly we next turn to computational modelling.”**

*c. Computational model: The authors are very technical in their description of the modeling approach. I think it is important to motivate the use of the model – and indeed the task itself – at a higher level. Relevant questions to answer are: What does the task measure and why do we care? Is there an example of where we might find its metrics useful to better describe/understand a disorder or similar? What do the different behavioural metrics tell us? What do the different computational parameters tell us? How do they relate to behaviour (on a theoretical level, but also maybe empirically)? (Some of this could go to the introduction). The technical description could also be improved. Not every variable in the model is properly explained (I cannot find an explanation of z, for example, or s). Likewise, technical descriptors should be defined (e.g., what is a tonic learning rate in this context? What is coupling strength?)*

Thank you for this comment. We have now added multiple clarifications throughout the paper to better convey higher level motivations and to provide more intuitive understanding of what the assay captures. In the introduction we clarify higher-level motivations:

“Here we investigate test-retest reliability of a computational assay probing advice-taking under volatility. The assay is comprised of a social advice-taking task and a Hierarchical Gaussian Filter (HGF) model (Diaconescu et al., 2014). **The task has been designed to probe how people infer the intentions of others (helpful vs. unhelpful) in the context where the intentions themselves are changing over time. This allows studying the dynamics of how people learn to trust or distrust others. Combining the task with the HGF model further helps to infer various cognitive variables of interest, in particular as it relates to participants’ estimation of how volatile the adviser’s strategy is and whether they are likely to be helpful or unhelpful at any given moment.** Variants of this assay have been used to study multiple disorders **that exhibit difficulties in correctly inferring the intentions of others**, including schizophrenia (Hauke et al., 2023; Henco et al., 2020), autism (Sevgi et al., 2020), and borderline personality disorder (Henco et al., 2020). Despite the use of the assay to study mental disorders, the reliability of the measures afforded by the assay has not yet been studied.”

We then describe all computational measures and motivate the need for computational modelling:

**“ 2.4 Behavioral measures**

**For the behavioral analysis we computed standard behavioral measures. This included: 1) Total accuracy, which was defined as the fraction of trials in which participants chose the correct response. This measure captures the overall level of performance. 2) Advice taking probability, which was defined as the fraction of trials in which participants acted in accordance with the social cue. This measure captures the tendency to rely on advice, and we computed it separately for stable and volatile phases of the task, as well as for the whole task. 3) Win-stay probability, which was defined as the fraction of trials in which participants kept the same strategy (trusting the advice or not) following a winning trial. 4) Lose-switch probability, which was defined as the fraction of trials in which participants switched their strategy (trusting the advice or not) following a losing trial. Both win-stay and lose-switch measures capture how quickly participants learn from positive and negative feedback, respectively. However, it is a rather rough proxy for the learning rate, because we expect it to dynamically change depending on how volatile the adviser’s strategy is perceived to be at any given point in the task – to capture these dynamics more explicitly we next turn to computational modelling.”**

We now clarify the meaning of s:

“with ***s* being a sigmoid function:**

“

Note that *z* here is simply a random variable (i.e., any input into the function).

We now also provide more intuitive and high-level explanations of each parameter in the caption of figure 2:

“Figure 2: Graphical representation of the mean-reverting 3-level HGF. The highlighted parameters are free parameters. **m_3_ is volatility equilibrium point towards which the participant slowly drifts irrespective of the experienced task outcomes. μ_3_^(0)^ and μ_2_^(0)^ are the priors about advice volatility and fidelity, respectively, that the agent has before starting the task. κ_2_ is the phasic learning rate which regulates how much the inferences about the volatility (at the third level) modulate learning about adviser’s fidelity (at the second level). ω_2_ is the baseline leaning rate (evolution rate), which regulates the learning rate at the second level independent from the estimates at the third level. ζ parameter captures how much participants rely on the social cue relative to the non-social cue. Finally, ν captures how noisily the beliefs about the correct choice are translated into action.**”

*d. Model space and model selection: It would be good to separate model space and model selection (which should come after model fitting?). The authors should also make sure they explain all acronyms (TAPAS, VBA, mHGF, sHGF). Further, it would help if the authors were a bit clearer in their prose. For example, what does it mean for a model to be “implemented using ‘tapas_hgf_binary’ function” (l.156-7)? What does the function do? Likewise, l. 159, what two hypotheses do the authors mean? They could either be stated explicitly, or the phrasing changed to “to arbitrate between the two models”.*

Thank you for these suggestions. We have now separated ‘Model selection’ section from ‘Model space’ section and moved it to appear after ‘Model fitting’ section.

“

**2.5.2	Model space**

In total, we have tested 2 variants of the HGF: with the mean-reverting process at the third level, Eq. 5 (model 1), and without, Eq. 4 (model 2). For each of these variants, we included an additional control model (model 1b and model 2b), in which all parameters of the perceptual model were fixed to parameter values of an ideal Bayesian observer optimised based on the inputs alone.

The models were implemented in Matlab R2020a using HGF 3.0 open-source toolbox included in Translational Algorithms for Psychiatry-Advancing Science (TAPAS) (Frassle et al., 2021) software collection https://github.com/translationalneuromodeling/tapas/releases/tag/v3.0.0). The code implementing the models can be found in tapas_hgf_binary.mfor model 1 and tapas_hgf_ar1_binary.m for model 2.

**2.5.3	Model fitting**

**Following the modelling pipeline in Hauke et al., (2024)**, meta-volatility ϑ was fixed to 0.5, and the drift rate parameter ϕ3 was fixed to 0.1**, which was done to reduce the number of free parameters and help with model convergence.** The remaining parameters were free, including: the evolution rate at the second level ω_2_, the coupling strength between the second and third level κ_2_, prior expectations before seeing any input about the adviser’s fidelity µ_2_^(0)^ and the volatility of the adviser’s intentions µ_3_^(0)^, the relative weighing of the advice compared to the non-social cue ζ, decision noise ν, and volatility equilibrium m_3_.

Model fitting **(i.e., finding parameter values with the highest posterior probability)** was done using quasi-Newton optimization algorithm. The priors of all model parameters were based on previous work, **the details of which can be found in Table 1 in Hauke et al., (2024). We briefly summarize them here in Table 1.**

**2.5.4	Model selection**

**To arbitrate among the models,** we performed random-effects Bayesian model selection (Rigoux et al., 2014; Stephan et al., 2009), using Variational Bayes Analysis (VBA) toolbox (Daunizeau et al., 2014) (https:// mbb-team.github.io/VBA-toolbox/). We present protected exceedance probabilities (PEP), which quantify the likelihood of a model being more plausible than any other model in the model space, while accounting for the possibility that model differences could arise by chance alone (Stephan et al., 2009; Rigoux et al., 2014). Additionally, we report relative model frequencies or f as an effect size measure, representing the probability that a randomly selected participant would be best accounted for by a specific model.

”

All the acronyms and technical terms have now also been clarified:

“The models were implemented in Matlab R2020a using HGF 3.0 open-source toolbox included in **Translational Algorithms for Psychiatry-Advancing Science (TAPAS)** (Fr¨assle et al., 2021) software collection https://github.com/translationalneuromodeling/ tapas/releases/tag/v3.0.0).”

“To arbitrate **among the models**, we performed random-effects Bayesian model selection (Rigoux et al., 2014; Stephan et al., 2009), using **Variational Bayes Analysis** **(VBA)** toolbox (Daunizeau et al., 2014) (https:// mbb-team.github.io/VBA-toolbox/).”

The ‘tapas_hgf_binary’ and ‘tapas_hgf_ar1_binary’ functions simply contain all the code implementing the models that we described in the text. We have now rephrased this to make it clearer:

“**The code implementing the models can be found in tapas_hgf_binary.m for model 1 and tapas_hgf_ar1_binary.m for model 2.**”

We now also use your suggested phrasing and refer to the ‘models’ rather than ‘hypotheses’ in the Model selection section:

“**To arbitrate among the models**, we performed random-effects Bayesian model selection…”

*e. Model fitting: The authors do not properly motivate why they fixed the meta-volatility and drift-rate parameters. Is there a conceptual reason (are those parameters not of interest? If so, why not?), or perhaps an empirical reason (models fail to converge)? The fitting procedure itself also deserves more attention. What is it that is optimised using the quasi-Newton optimisation algorithm? I assume a posterior likelihood, given that there are priors involved? This should be stated. The priors themselves should be presented in the main body of the article – priors can have important consequences for reliability. Did the authors consider using multivariate priors (i.e., priors that include correlations between parameters?)? If not, why not?*

The meta-volatility and drift-rate parameters were fixed following the modelling pipeline in Hauke et al., (2024). The original reasons for fixing these params included: the need to reduce the number of free parameters to improve the convergence and stability of the model, and choosing to fix parameters that we did not have specific hypotheses for. A more detailed explanation for how the specific values for selected can be found in the original paper, with Table 1 summarizing the previous literature that the values were based on:

We have now clarified what we mean by the model fitting procedure:

“Model fitting **(i.e., finding parameter values with the highest posterior probability)** was done using quasi-Newton optimization algorithm.”

We have now clarified in the text where to find more information about the priors and have moved the table to appear in the main text:

“**The priors off** **all model parameters were based on previous work, the details of which can be found in Table 1 in Hauke et al., (2024). We briefly summarize them here in Table 1**.

Using multivariate priors that capture correlations among parameters were not considered. Implementing multivariate priors adds substantial complexity to the analysis and the benefits are not guaranteed, especially when we found no significant collinearity among the parameters. Using univariate priors is simpler, more robust to misspecification, and can provide more stable estimates given the same amount of data.

*2.Results
a.Parameter recovery vs test-retest reliability:*

*i. The authors imply that the ICC between true and recovered parameters reflects (inverse) measurement error. My understanding is that parameter recovery primarily assesses a model's capacity to accurately reflect behaviour. That is, whether a specific set of parameter values uniquely maps onto a specific behavioural pattern or whether this pattern can also be achieved using a different set of parameter values. This can be understood to reflect (inverse) measurement error insofar as the model parameters are taken as instruments measuring behaviour? Is that what the authors mean? It would be great if they could provide further clarification on this point.*

Yes, that is correct!

We have now amended the description of parameter recovery to incorporate your wording, which hopefully will make things clearer for an average reader:

“Parameter recovery involves generating synthetic data of task performance using a range of model parameter values and then fitting the same model to that data (Wilson and Collins, 2019; Palminteri et al., 2017). **This procedure allows us to assess how uniquely different sets of parameter values map onto specific behavioral patterns, and vice versa.** The similarity between the ’true’ and the ’recovered’ parameters indicates how reliable the estimates would be under ideal conditions: when task performance stays exactly the same across instances.”

“As such, **it is constrained only by the intrinsic measurement** error and can be considered to provide an upper bound on test-retest reliability, **because the latter would be further affected by within-subject changes in task performance** (Karvelis et al., 2023).”

*ii. I wonder if the authors have considered comparing the test-retest reliability to an internal consistency measure rather than (or better, in addition to) the parameter recoverability? The difference between the two should relatively straightforwardly map onto state changes, right?*

Internal consistency measures, such as split-half reliability, can only be meaningfully applied to tasks that have independent trials. Our task, on the other hand, depends on trial-by-trial learning, therefore the trials cannot be separated to compute internal consistency measures.

*iii. That said, I think the fact that some model parameters recover poorly receives too little attention (both in the results and the discussion). As the authors say, it provides an upper bound to their reliability (both internal consistency and test-retest) and is therefore absolutely crucial for reliability. Was the recoverability of those parameters also poor in previous applications of the task+model? Is this because some of the parameters trade off against each other? Could this be remedied by (a) finding the optimal number of free parameters in a more extensive model selection procedure, and/or (b) using a multivariate prior that includes correlations between parameters? If not, is there an alternative model that may have better recovery properties? If not, do we have to conclude that this task+model is unsuitable for interindividual difference research altogether?*

Parameter recovery that we find in this study is comparable to what has been found before (e.g., Hauke et al., 2024; Diaconescu et al., 2020). It should be noted, however, that previous research did not have clear criteria for what should be considered ‘good’ parameter recovery – this applies not just to this assay, but to research in computational psychiatry more broadly. In previous research, ~ 0.5 correlation between true and recovered parameters would be considered as showing very good parameter recoverability, as per the conventional labelling of 0.5 correlations as being ‘large’. By introducing the view of parameter recovery as the upper bound on reliability in our previous work (Karvelis, et al., 2023), we have made the criteria for what should be considered good parameter recoverability much clearer and stricter.

We now add this clarification in the discussion:

“**The first thing to ensure in task optimization is high parameter recoverability. Here we found that some parameters were recovered poorly, making it impossible to have high test-retest reliability for these parameters. Note that parameter recoverability of ~0.5 would not have been considered too problematic in previous research, as it would be interpreted as a ‘large’ effect size. However, considering parameter recoverability as the upper limit for test-retest reliability (Karvelis, et al., 2023) imposes much stricter criteria for what recoverability can be considered satisfactory.”**

Why parameter recovery is low for some parameters is not easy to determine. To assess the possibility of collinearity, we have now done some further analysis by fixing poorly recovered parameters one by one to check if it would improve the recovery of the remaining parameters (Fig 5 in S1 Appendix). This analysis suggested that poor recovery was not due to collinearity, as fixing one parameter did not improve recovery in other parameters. Another potential way to improve parameter recovery is to increase the number of trials. However, because our task depends on trial-by-trial dynamics, it is simultaneously a question of what advice probability structure should be chosen for the extra trials and for the whole task overall (e.g., how should the volatility of the advisor change over time) – it is difficult to find a principled way to address this challenge. Finally, one more easily addressable factor that may be limiting parameter recoverability is the binary nature of responses in this task (Mikus et al., 2023). Future studies should consider incorporating continuous response measures, which contain more information and can therefore constrain parameter estimates better

“**How the parameter recoverability itself could be improved does not have a straightforward answer. Sometimes low recoverability of parameters stems from collinearity – when parameters trade-off among each other to provide equally good fit to the data. Here we ruled out this possibility by fixing poorly recovered parameters one by one and showing that it did not improve recoverability of the remaining parameters (Fig 5 in S1 Appendix). Another potential way to improve parameter recovery is to increase the number of trials. However, because our task depends on trial-by-trial dynamics, it immediately reverts us back to a more question of what advice probability structure should be chosen (e.g., how should the volatility of the advisor change over time). It is likely that a different structure than was used in this task could constrain the parameter estimates better – however, discovering such structure in a principled way is a rather challenging problem. Finally, one more easily addressable factor that may be limiting parameter recoverability is the binary nature of responses in this task (Mikus et al., 2023). Future studies should consider incorporating continuous response measures, which contain more information and can therefore constrain parameter estimates better.**”

Finally, the poor test-retest reliability of the current model parameters does not necessarily make it unsuitable for individual differences research. However, it does require considering that any observed effects will be strongly attenuated – in this context the interpretation should be done on parameter-by-parameter basis, which is what we did in the discussion by considering the findings by Hauke et al., 2024 concerning parameters κ_2_ and μ_3_^(0)^_._

*b. Practice effects: Please explain, for each measure, what an increase or decrease means on a psychological level and why.*

We have now added this to section “3.5 Practice effects”:

“Among the computational measures, only µ(0) and ζ estimates were significantly different between the sessions. **Both parameters were found to be increased in the second session, indicating that participants expected the adviser to be more helpful before starting the second session (μ_2_^(0)^), and tended to rely on the social cue more throughout the task (ζ).** For all other parameter estimates, there was substantial evidence (BF_01_ > 3) for there being no practice effects.”

*c. Face validity: Have the authors considered moving this section to the beginning of the results section? This would also provide an opportunity to explain the model and its parameters at a higher level and in relation to task behaviour.*

Thank you for the suggestion. We decided to keep the section where it is right now because it relies on findings presented in 3.5, where we determine which parameters have significant practice effects, which in is motivated by findings in 3.4 where we investigate test-retest reliability of computational measures.

We hope that the additional high-level explanations that we provided in response to your other questions will be already sufficient to clarify the basic features of the model.

*3.Discussion:
a. Improving test-retest reliability: I believe the authors may have partially misunderstood the analyses by Brown et al. / Waltmann et al. The parameter estimates derived from jointly modelling data from the two sessions should actually be more accurate than those derived by separate modelling of the data. This should be true even though (in fact because) partial pooling biases the estimates towards the within subject and between subject means. Perhaps counterintuitively, the estimates are, on average, closer to the ground truth than unbiased estimates (i.e., estimates that do not use partial pooling across sessions or individuals) (see Effron and Morris’ 1979 paper "Stein’s Paradox in Statistics" for a detailed explanation on how and why this is possible; also see Rouder & Haaf for a more contemporary treatment). As the authors correctly point out, an issue arises when one tries to calculate ICCs from these estimates – these will be inflated. Indeed, calculating ICCs from the estimates may violate the assumptions of the ICC because due to partial pooling, the estimates are no longer independent from one another (this messes with the variances – although, as the authors also state, Brown et al. and Waltmann et al. found a way to circumvent this problem). However, whether the ICCs (i.e., the reliability metrics) are biased or not has no bearing on the accuracy of the parameter estimates themselves – per Stein’s Paradox, they should still be better.*

Thank you for this clarification. We have now updated our discussion to incorporate these observations.

“A simulation analysis by Waltmann et al. (2022) has demonstrated that estimating reliability this way is more accurate compared to modelling the sessions separately. However, computing ~~r~~**~~eliability~~ ICCs** using parameter estimates obtained from the joint modelling of the sessions resulted in inflated reliability (Waltmann et al., 2022).~~, indicating that the parameter estimates themselves were biased to be more similar between the sessions than they actually were.~~ **It means that using these point estimates in further analyses without accounting for their uncertainty and their non-independence would also introduce biases**. Given that we are **primarily** interested in the parameter estimates **themselves** rather than **only** the model-derived estimate of reliability, it is unclear how practical this approach is. For this reason, we stuck with a simpler model-fitting approach and modelled each session separately (see Methods for details).”

*b. Validity: As in the results section, I would move this to the top of the discussion.*

We kept it as it is, to be consistent with the results section.

*c. Limitations: The sample size is very small. I understand that this is because the data is derived from a clinical study and I am glad the authors still put together this paper but it should be clearly stated as a limitation. The same goes for the difference in the context in which the task was performed (EEG vs fMRI) – this is a source of variance that would be controlled in a more targeted reliability study. Even if the authors show that there is no bias (no directed, fixed effect) the change in context is almost certain to introduce (nonspecific) error variance.*

Thank you for this suggestion. We have now added a limitations section where we address the concerns that you raised:

“**4.5 Limitations**

**Our sample size in this study was relatively small (N = 39), which resulted in rather wide confidence intervals around the estimated mean ICC values and outsized influence of individual datapoints in some cases (e.g., for κ_2_ reliability estimates). While there is no consensus for the optimal sample size for reliability studies - the suggested optimal sample sizes range from ∼ 60 (Williams et al., 2023), to 100 (Kennedy, 2022), to 400 subjects (Charter, 1999) - larger samples can provide more precise reliability estimates, which is especially important when reliability itself is poor. Another limitation in our study is the fact that our test and retest sessions were done during EEG and fMRI scanning. Even though we showed that there were no fixed effects on task performance between the two experimental environments, it may have nonetheless introduced additional random effects and contributed to the error variance.**“

*d. Conclusion: This could be more task (and perhaps even parameter) specific – what did the authors find and what does it mean for using the task in the future?*

We have now added a sentence summarizing assay-specific findings (note that we provide a parameter specific discussion in 4.2):

“**Our findings of a largely poor test-retest reliability of behavioral and computational measures of advice taking under volatility warrant more caution when interpreting previous findings of this (Hauke et al., 2024) and potentially other versions of the assay (Henco et al., 2020; Sevgi et al., 2020; Reiter et al., 2021). More generally,** our findings add to the growing body of research showing that many behavioral and computational task measures suffer from poor test-retest reliability (Karvelis et al., 2023; Enkavi et al., 2019; Hedge et al., 2018). We sought to illustrate how using simple methods, one can start to disentangle the different sources of variance underlying low test-retest reliability of computational measures, however, more intentional experimental designs are needed for a more in-depth analysis of these sources (e.g., Schurr et al., 2023). A better understanding of what factors drive low test-retest reliability would facilitate the design of more psychometrically sound assays, which would increase the quality of future research and the possibility of clinical translation.“

*Minor:
1. The abstract could be more concrete/specific and more assertive in tone.*

We are not exactly unsure what the reviewer had in mind with being more concrete/specific (we clearly state both qualitative and quantitative results) or by being more assertive (we clearly state the implications of our findings and the growing need to address these issues in this area of research). Note that the poor performance of the assay is not specific to this assay and reflects a more general problem in the field. That is why in the abstract we highlight methodological aspects and more general implications of our findings, rather than the implications of these findings for a handful of previous studies using this assay.

*2. In the beginning of the introduction, the authors suggest that there is growing enthusiasm that computational measures may become a new basis for psychiatric nosology. Given that no interventions (that I know of) have flown from almost two decades of computational psychiatry research, and (to my knowledge) no unequivocal mappings of computational parameter alterations to symptoms have emerged, I think this is a bit of an overstatement – I rather feel like the field is waking up to its limitations (such as poor reliability)!*

We have now changed “There is a growing enthusiasm…” to “**There is plenty of enthusiasm…**” to be more precise about the sentiment in the field. Note that the most impactful recent review reviews that we cite to support the “enthusiasm” statement primarily focus on the clinical potential and discuss psychometric issues only briefly if at all. Our recent review paper (Karvelis et al., 2023) is the only one that aimed to document the current limitations in detail. The sentiment at the computational psychiatry symposia and conferences also tends to be largely optimistic and the psychometric issues still do not receive enough attention.

*3. L. 48 ff: I would suggest moving the periods and commas around to make this more effective: “Up until recently, the psychometric properties of computational measures have received little attention (Browning et al., 2020). Emerging empirical evidence indicates that both reliability and construct validity are often poor (Karvelis et al., 2023) but the psychometric properties of many currently used computational assays remain unknown...”*

Thank you for this suggestion, we have now adjusted this paragraph as you suggest.

*4. L. 230: “<¡0.8”, is this a typo?*

Thank you for spotting this. It has now been corrected to “< 0.8”

*5. L. 252: Do the authors mean “i.e.,” rather than “e.g.,”?*

Thank you for spotting this. It has now been corrected to “i.e.”.

*6. L.256-7: Do the authors mean an increase across sessions? It’s best to be precise here.*

We have now clarified this:

“Among the behavioral measures we found significant increases **during the second session** in advice-taking during the stable phase, close to significant increases in advice-taking during the volatile phase, significant increases in advice-taking overall, and increased win-stay probability (Fig 6).”

*7. L. 344 ff: This paragraph comes a little out of the blue – can it be integrated with the previous paragraph?*

Thank you, we have not connected this to the previous paragraph.

*8. L.391: Citation is missing brackets.*

Thank you, this has now been corrected.
