# Supplementary material for: Test-retest reliability of behavioral and computational measures of advice taking under volatility
Source: PLoS One. 2024 Nov 18;19(11):e0312255. doi: 10.1371/journal.pone.0312255 (PMC11573178; doi:10.1371/journal.pone.0312255)
Supplement: Attachment — Submitted filename: Response to reviewers - 2.docx [file pone.0312255.s003.docx]

**“Test-retest reliability of behavioral and computational measures of advice taking under volatility”**

**Responses to reviewers – 2^nd^ round**

We would like to thank the reviewer for an opportunity to provide additional clarifications!

*Reviewer #2: The authors have answered almost all of my queries satisfactorily and I congratulate them on their hard work. I particularly appreciate their higher level explanations that make the manuscript much more accessible. I only have two minor queries regarding their responses.*

*2.a.i. Regarding internal consistency, I understand that it is hard to separate odd and even trials in a learning task, but it is possible to compare parameters derived from the first and second halves of a task. I know that this has its own limitations but I think it might be helpful to at least get a rough idea of how much variance between sessions might be due to state changes vs noise.*

Thank you for this suggestion. In our case, the split-half reliability measures for this task would still not be informative no matter how we split the trials (odd vs even or first vs second half). The first half of the task is not the same as the second half of the task in terms of the reward probability structure – so even in theory, we would expect the behavior and the resulting parameter estimates to differ between the two halves of the task. This would appear as noise, but only because we are comparing different stages of the task (e.g., stable versus volatile). This would be equivalent to computing internal consistency among different conditions in other task paradigms – the conditions are meant to induce different behaviors, but the difference between them has no information about how good overall test-retest reliability is going to be or what the sources of low reliability would be.

*3.a. As I mentioned in my previous comment, point estimates derived from hierarchical models are, on average, more accurate than those from flat models (literature refs as before). The fact that they are not independent from one another does not explain, by itself, why using them in further analyses should introduce bias, and in what way or what direction. It would be good if the authors could explain their reasoning in more detail here. Moreover, even if using these estimates did introduce bias, "accounting for their uncertainty and their non-independence" in further analyses as the authors suggest is not difficult – it can be done by simply including the additional variables of interest in the model – and therefore should not, in principle, be too much to ask if hierarchical modelling conveys a significant advantage in estimation accuracy. Is there any particular use case that the authors envision in which this would be unreasonable or impossible?*

Our concern arises from the use of jointly estimated point estimates to compute intra-class correlation coefficients (ICCs), which results in inflated values. As you noted in your previous response, this inflation occurs because the computation violates the assumptions of independence that the ICC relies on.

Similarly, Pearson's correlation and regression, which would be most likely employed in subsequent analyses (see below), also rely on the assumption of independence. Therefore, we anticipate encountering similar problematic biases in these methods.

Some examples of most likely further analyses would include: 1) correlating point estimates with external measures (e.g., symptoms, personality traits, measures from other tasks) to study convergent and clinical validity, or 2) correlating the differences between the two time points in these measures to study longitudinal validity.

This means that researchers would need to use more sophisticated hierarchical modelling techniques (that incorporate information about the full posterior distribution, not just the point estimates) in further analyses. It is certainly doable, but less practical due to the additional complexity.

We now address this in the main text in a more nuanced way. We also include a comment on the idea of implementing the joint modelling approach for HGF modelling.

“Recent work has demonstrated that modelling task performance jointly across the two testing sessions can improve reliability (Brown et al., 2020; Sullivan-Toole et al., 2022; Waltmann et al., 2022). In this approach, each parameter is estimated in a form of a bivariate distribution, from which a correlation coefficient between the sessions can be derived. A simulation analysis by Waltmann et al. (2022) has demonstrated that estimating reliability this way is more accurate compared to modelling the sessions separately. However, computing ICCs using parameter estimates obtained from the joint modelling of the sessions resulted in inflated reliability (Waltmann et al., 2022). It means that using these point estimates in further analyses without accounting for their uncertainty and their non-independence would also introduce biases. ~~Given that we are primarily interested in the parameter estimates themselves rather than the model-derived estimate of reliability, it is unclear how practical this approach is. For this reason, we stuck with a simpler model-fitting approach and modelled each session separately (see Methods for details).~~ **To avoid these issues, future analyses should utilize full posterior distributions rather than relying solely on point estimates. Nonetheless, here we used a simpler modelling approach that relies on fixed priors (see Methods for details), as this is the only established optimization pipeline available for fitting HGF models (Mathys et al., 2014). Joint modelling of the sessions - or even modelling the sessions separately but with empirical priors – poses significant challenges for the HGF. Without fixed priors, there is less control over regularization, increasing the risk of parameter values falling into regions that violate model assumptions and causing the optimization to fail. These limitations will be addressed in future work.**

**While hierarchical modelling approaches may provide some improvement in the reliability of parameter estimates, f**ocusing on task design optimization seems to be a much more promising avenue **as it can address the root causes of low reliability.** ~~for improving reliability~~. Most cognitive tasks have been optimized for producing strong group effects, which often means low between-subject variation and as a result, low test-retest reliability (Hedge et al., 2018)….”
