# Supplementary material for: Test-retest reliability of behavioral and computational measures of advice taking under volatility
Source: PLoS One. 2024 Nov 18;19(11):e0312255. doi: 10.1371/journal.pone.0312255 (PMC11573178; doi:10.1371/journal.pone.0312255)
Supplement: Attachment — Submitted filename: Response to reviewers - 3.docx [file pone.0312255.s004.docx]

**“Test-retest reliability of behavioral and computational measures of advice taking under volatility”**

**Responses to reviewers – 3^rd^ round**

Thank you for the additional comments and for providing clarification on how to address them. Below we provide our responses. The original comments are in *italic*, our responses are in regular font, while cited changes in the manuscript are indicated in **bold**.

*- Grammar: I recommend carefully reviewing the Abstract and Introduction, focussing on*

*simplifying complex sentences for better clarity. For example, in the Abstract, the sentence "The*

*research significantly advances our understanding of rapidly evolving trends in..." could be*

*rephrased as "This research advances our understanding of fast-changing trends in..." —*

*removing unnecessary adverbs like "significantly" and "rapidly" to make it more concise.*

*Additionally, restructuring longer sentences in the Introduction will help avoid redundancy and*

*improve readability.*

Thank you for this suggestion. The sentence you are referring to does not exist in our abstract but we have reviewed our manuscript for any possible grammatical improvements.

*- Refs: I agree that the refs need to be adjusted to adhere to the Journal’s guidelines (c.f.*

*https://journals.plos.org/plosone/s/submission-guidelines). Refs should be numbered in square*

*brackets and listed in the order they appear in the text. Currently, they are cited by name, which*

*deviates from the standard practice.*

*For example, your references are formatted slightly differently from the Vancouver style:*

*- Balter, L. J., Matheson, G. J., Sundelin, T., Sterzer, P., Petrovic, P., and Axelsson, J. (2022).*

*Experimental sleep deprivation results in diminished perceptual stability independently of*

*psychosis proneness. Brain Sciences, 12(10):1338.*

*This should be corrected throughout the ms to ensure compliance. "References are listed at the end of the manuscript and numbered in the order that they appear in the text. In the text, cite the reference number in square brackets (e.g., “We used the techniques developed by our colleagues [19] to analyze the data”). PLOS uses the numbered citation (citation-sequence) method and first six authors, et al."*

Thank you for pointing this out. We have now reformatted the citations from APA style to numbered, and changed the references to be in Vancouver style, in accordance with the PLOS ONE guidelines. Note these changes are not tracked in the provided manuscript because we reformatted it in latex. All other changes are tracked.

*- Tables: I am unsure if this comment requires a rebuttal. However, I did notice some*

*inconsistency in reporting the parameters. Additionally, if this Table is reproduced from another*

*source, please ensure that you have the necessary permissions and disclose this in the ms.*

*Overall, the Table needs some refinement to maintain consistency in data presentation and to*

*clearly place abbreviations for the parameters.*

We have now updated the table so that the column names directly refer to the actual parameters - which makes the table more concise and clearer.

*- Clarity on the graphs: I understand the revs' comments, which mainly address avoiding clutter.*

*The diagram, as mentioned, needs some refinement to be clearer and more straightforward. This*

*also applies to the font sizes and the comment on 'consistency.' I suggest increasing the font size*

*of both the axes and captions to improve readability. Additionally, consider adding a brief*

*explanatory note in the caption to detail the dashed lines, coefficients, and other important*

*parameters.*

Thank you for this suggestion. We have gone through all the figures and made sure that the font sizes of all axes are at least larger than the font size of the main text – if the main text is readable, the figures will definitely be readable.

*2-) After re-reading the revs' comment, I believe the sample size and number of trials in your*

*study are sufficient for robust analysis. It seems that your model is robust and consistent with*

*other studies (c.f. 10.1037/met0000275 or Wagenmakers' studies. In fact, these studies could be*

*very useful for providing additional information on priors and posteriors.*

The certainty of the results that we get with the given sample size and the number of trials is directly reflected in the ICC confidence intervals that we report for each measure. Larger sample sizes would simply give us narrower confidence intervals and more certainty around the mean estimate of ICC. Note that if the ICCs were much higher (e.g., ICC > 0.9), even with the current sample we would get much narrower confidence intervals (as can be seen in the parameter recovery analysis). This means that even with the current sample we can be confident that ICC of behavioral and computational measures for this assay are rather low despite some uncertainty around their exact values.

Thank you for referring to additional studies related to this. While these studies are methodologically relevant, they cannot provide direct information for the priors that we use because we are using different types of models.

*3-) I spent some time reading on the revs' remaining comments, particularly re. the flexibility of*

*your model. It seems the rev may have misunderstood your rationale for fixing certain*

*parameters. While your intention to avoid overinflating the model is clear, it'd be helpful to*

*provide a brief explanation in the text about why some parameters were fixed while others were*

*allowed to vary. This would address concerns about potential oversimplification or overfitting.*

*Referencing previous studies that demonstrate the stability of these parameters could further*

*support your argument.*

*Fixed parameters: The concern about fixed parameters and potential overfitting is important. I*

*suggest adding a brief explanation of why certain parameters were fixed and how this decision*

*helps prevent overfitting. You might reference previous studies (e.g. Wagenmakers et al.) that*

*used a similar approach to provide justification. Additionally, clarifying your choice of priors*

*would help address this concern.*

Model complexity and which parameters are fixed is indeed an important consideration. If not sufficiently explored, this indeed can lead to overfitting and results that do not generalize. That’s why in our analysis we performed an extensive exploration on how model complexity and different number of parameters affect our results – what we found is that computational measures showed similarly poor reliability across all of these models.

For example, we report test-retest reliability results not just for the winning model 2, but model 1. See supplementary figure 4:

Furthermore, in the context of investigating collinearity, we perform extensive analysis of how fixing different parameters affect test-retest reliability of the remaining parameters. We find that the overall test-retest reliability stays very similar no matter which parameters are fixed.

In the discussion we write:

“How the parameter recoverability itself could be improved does not have a straightforward answer. Sometimes low recoverability of parameters stems from collinearity – when parameters trade-off among each other to provide an equally good fit to the data. Here we ruled out this possibility by fixing poorly recovered parameters one by one and showing that it did not improve recoverability of the remaining parameters (Fig 5 in S1 Appendix).”

And in the appendix we provide the results:

To summarize, model complexity and which parameters are chosen to be fixed, does not lead to substantial differences in the overall test-retest reliability of the computational measures. That means that the conclusions we make in our paper do not hinge on one specific implementation of the model.

As for the justification of priors for each parameter, in the text we refer to our previous study (Hauke et al., 2024) that contains detailed justification:

*This is extende to learning effects: The debate on learning effects has been largely debated.*

*However, it would be beneficial to include a brief discussion in your text on how learning effects*

*might influence your results (i.e. if possible), particularly in a test-retest context. Scharfen’s*

*studies on test-retest reliability or Salthouse’s work could serve as useful references. A short*

*sentence acknowledging this potential limitation could be added to the Discussion.*

Considering learning effects is definitely very important. Our manuscript already presents and extensive analysis of learning effects. In section 3.5 Practice effects we analyze learning effects on both behavioral and computational measures. We do find significant effects for some parameters, but we find that it has rather negligible effect on test-retest reliability. In this section we write:

“It is interesting to note that while for both μ_2_^(0)^ and ζ, practice effects were of moderate size

(Cohen’s d = 0.37 and d = 0.42, respectively), its contribution to low test-retest reliability of these

parameters was minimal, reducing it only by 0.02 and 0.03, respectively, as indicated by the differences

between the absolute agreement ICC and Pearson’s correlation (Fig 4).”

Note that we also already discuss this in the discussion:

“We considered three sources of within-subject variance: practice effects, state-like fluctuations, or trait- like changes. We did find significant practice effects in some parameters, and while these effects were of moderate size (Cohen’s d = 0.37 - 0.42), they accounted only for a small fraction (ICC ≈ 0.02 − 0.03) of the reduction in test-retest reliability. Together with other studies reporting small to medium practice effects [33, 34], it suggests that fixed practice effects might be a fairly minor factor behind low test-retest reliabilities of computational assays (although see [35] for a more extensive analysis). Nonetheless, practice effects still remain understudied and underreported [9].”

We also already comment on this result in the abstract:

“Further analysis revealed that reliability was substantially impacted by intrinsic measurement noise (indicated by parameter recovery analysis) and to a smaller extent by practice effects.”

*Recoverability: The recoverability of your model appears adequate based on the evidence*

*provided. However, it may be helpful to briefly mention this in the limitations section, noting any*

*challenges that could arise in other studies involving more complex data.*

Indeed, there are many considerations to keep in mind when it comes to recoverability. We address these in the discussion section:

“The first thing to ensure in task optimization is high parameter recoverability. Here we found that some parameters were recovered poorly, making it impossible to have high test-retest reliability for

these parameters. Note that parameter recoverability of ∼ 0.5 would not have been considered too problematic in previous research, as it would be interpreted as a ‘large’ effect size. However, considering parameter recoverability as the upper limit for test-retest reliability [9] imposes much stricter criteria for what recoverability can be considered satisfactory.

How the parameter recoverability itself could be improved does not have a straightforward answer. Sometimes low recoverability of parameters stems from collinearity – when parameters trade-off among each other to provide an equally good fit to the data. Here we ruled out this possibility by fixing poorly recovered parameters one by one and showing that it did not improve recoverability of the remaining parameters (Fig 5 in S1 Appendix). Another potential way to improve parameter recovery is to increase the number of trials. However, because our task depends on trial-by-trial dynamics, it im- mediately reverts us back to a more general question of what advice probability structure should be chosen (e.g., how should the volatility of the advisor change over time). It is likely that a different structure than was used in this task could constrain the parameter estimates better – however, dis- covering such structure in a principled way is a rather challenging problem. Finally, one more easily addressable factor that may be limiting parameter recoverability is the binary nature of responses in this task [32]. Future studies should consider incorporating continuous response measures, which contain more information and can therefore constrain parameter estimates better”

*Cross-validation: I understand revs' comment, which appears to reflect concern over potential*

*issues. However, the ms is clear on this point. If time allows, please consider providing*

*information on posterior predictive checks and how they could strengthen the robustness of your*

*model. If not, a brief sentence explaining the trade-offs and why posterior checks were not*

*conducted would be useful.*

Cross-validation would definitely be a promising alternative to the current model comparison methods in computational psychiatry. However, our sample is not large enough to implement unbiased cross-validation approaches. Even more importantly, this would not have any effect on our conclusions because we already report test-retest reliability not only for the winning model 2, but also for model 1, as well as many other models when we explore the consequences of fixing different model parameters.

*Self report bias: Consider briefly mentioning the possibility of self-report bias, as it is common.*

*You could add a sentence in the limitations section stating that, while self-report bias may exist,*

*your assumptions remain robust and are supported by external validation checks.*

Self-reporting bias considerations apply in the context when self-report measures are analyzed. In this study our analysis was performed on task performance, which is not a self-report measure, hence self-reporting bias does not apply.

*Other comments: It would be beneficial to briefly explain how the learning rates in your model*

*are adaptive, as this may address the remaining concerns. A short paragraph on your choice of*

*Gaussian distributions could also help clarify why this approach was taken. Providing a clear*

*explanation of how the algorithm reaches the global maximum (or optima) would be useful in*

*reinforcing the reliability of your model.*

The foundations of how HGF is implemented are described in detail in previous literature that we refer to in the text:

“To model participants’ behavior in the task we used the same analysis pipeline as in the previous work [11], using a 3-level Hierarchical Gaussian Filter (HGF) [16, 17]. This modelling framework assumes that participants infer a hierarchy of hidden states (x1, x2, and x3) that characterize outcome probabilities and their temporal dynamics. The participants’ beliefs about the hidden states at each level i on trial k are denoted as µ(k).”

Note that we are not choosing Gaussian distributions, we are simply describing how this modelling framework is implemented. Gaussian distributions are essential for deriving update equations. This means there is no alternative implementation to consider, unless we want to introduce a new modelling framework – but this would be a big project way beyond the scope of this paper.

The model is fitted using quasi-Newton optimization algorithm, which is one of the standard optimization algorithms and we refer to it in the text:

“Model fitting (i.e., finding parameter values with the highest posterior probability) was done using quasi-Newton optimization algorithm.”

*It’s important to clarify technical concepts like collinearity and the choice of Pearson correlation*

*for readers outside of your immediate field. For instance, collinearity can sometimes confuse*

*those unfamiliar with regression analysis, so including a brief explanation of how collinearity*

*was assessed and why it matters to your results would be helpful.*

Indeed, it’s important to clearly communicate what these concepts mean. In the section 2.6 we explain Pearson correlation and its relevance in the context of our study:

“Alongside ICCs, we also report Pearson’s correlation coefficients in order to highlight when and how much these metrics diverge. Pearson’s r is not sensitive to offset and scaling errors, which makes it a less suitable metric for assessing reliability [9]. However, as we will see, in practice this might be rarely a problem. Furthermore, we will use the comparison between Pearson’s r and ICC to gauge the impact of practice effects on test-retest reliability.”

We also provide an explanation on why collinearity matters and how it was assessed in section 3.4:

“We also investigated whether the low parameter recoverability for some parameters (µ_2_^(0)^, µ_3_^(0)^ and

κ_2_) was due to collinearity among parameters. To test this, we fixed these parameters to their prior

values one by one and examined if it improved the recovery of the remaining parameters. We found that it did not, suggesting that collinearity was not the reason behind low recoverability (see Fig 5 in S1 Appendix for details).”

We provide further clarification on collinearity in the discussion section:

“How the parameter recoverability itself could be improved does not have a straightforward answer. Sometimes low recoverability of parameters stems from collinearity – when parameters trade-off among each other to provide an equally good fit to the data. Here we ruled out this possibility by fixing poorly recovered parameters one by one and showing that it did not improve recoverability of the remaining parameters (Fig 5 in S1 Appendix).”

*------------------------------------------------------------------------------------------------------------------------------*

Additional minor changes to the manuscript

Note that we found a small mistake in how we defined total accuracy in our task and have now corrected it. This does not change the overall interpretation of our findings.

Section 2.4 is now updated:

“For the behavioral analysis we computed standard behavioral measures. This included: 1) Total accuracy, which was defined as the fraction of trials in which participants chose the ~~correct response~~ **option with the** **higher reward probability**.”

And section 3.5 is now updated:

“First, we analyzed test-retest reliability of standard behavioral measures (Fig 3). We found all most measures to be in the poor reliability range (ICC < 0.5), **except for total accuracy (ICC = 0.61) and** win-stay ~~which was slightly higher~~ (ICC = 0.52). Comparing both absolute ICC and Pearson’s correlation for each of the measures also revealed that both indices provide similar results (with the largest differences being 0.0**3**).”

Figure 3A was also now updated:
